# Stability of anterior open bite treatment with molar intrusion using skeletal anchorage: a systematic review and meta-analysis

**DOI:** 10.1186/s40510-020-00328-2

**Published:** 2020-09-05

**Authors:** Daybelis González Espinosa, Paulo Eliezer de Oliveira Moreira, Amanda Silva da Sousa, Carlos Flores-Mir, David Normando

**Affiliations:** 1 Department of Orthodontics, Faculty of Dentistry, Dental School, Federal University of Pará (UFPA), Augusto Correa St., no. 1, Belém, Pará 66075-110 Brazil; 2 Facultad de Odontologia, Universidad Católica Redemptoris Mater, Managuá, Nicaragua; 3 Faculty of Medicine and Dentistry, University of Alberta, Edmonton, Canada

**Keywords:** Open bite, Skeletal anchorage, Molar intrusion, stability

## Abstract

**Objectives:**

The aim of this systematic review and meta-analysis is to assess the degree of stability of anterior open bite (AOB) treatment performed through the molar intrusion supported with skeletal anchorage at least 1 year posttreatment.

**Methods:**

This study was registered in PROSPERO (CRD42016037513). A literature search was conducted to identify randomized (RCT) or non-randomized clinical trials based including those considering before and after design. Data sources were electronic databases including PubMed, Cochrane Library, Science Direct, Google Scholar, Scopus, Lilacs, OpenGrey, Web of Science, and ClinicalTrials.gov. The quality of evidence was assessed through the JBI tool and certainty of evidence was evaluated through the GRADE tool. Random effects meta-analysis was conducted when appropriate.

**Results:**

Six hundred twenty-four articles met the initial inclusion criteria. From these, only 6 remained. The mean posttreatment follow-up time was 2.5 years (SD = 1.04). The overbite showed a standardized mean relapse of − 1.23 mm (95% CI − 1.64, − 0.81, *p* < 0.0001). Maxillary and mandibular incisors presented a non-significant mean relapse, U1-PP − 0.04 mm (95% CI − 0.55, 0.48) and L1-MP − 0.10 mm (95% CI − 0.57, 0.37). Molar intrusion showed a relapse rate around 12% for the maxillary molars and a 27.2% for mandibular molars.

**Conclusion:**

The stability of AOB through molar intrusion using TADs can be considered relatively similar to that reported to surgical approaches, since 10 to 30% of relapse occurs both in maxillary and mandibular molars. The level of certainty ranged between very low and low. RCTs reporting dropout during the follow-up are in dire need.

## Introduction

Anterior open bite (AOB) can be corrected by orthodontic extrusion of the anterior teeth, by surgical impaction of the maxilla in adult patients, or by the control of molar eruption in growing patients [1–3]. With the advent of skeletal anchorage, correction through orthodontic intrusion of the posterior teeth using titanium miniplates or monocortical bone screws became viable [4–7]. Skeletal anchorage, by promoting the intrusion of molars into their bony support, facilitates an anti-clockwise rotation of the mandible with the consequent bite closure. These effects are considered somehow equivalent to those of a maxillary impaction through orthognathic surgery [8].

Currently, there is no consensus on whether surgical or non-surgical treatment is the most stable approach for AOB treatment in adult patients. The associated stability or lack of thereof is under the influence of several factors, especially those AOB etiological factors [8]. Among those, tongue posture and size, persistence of digital sucking habits, respiratory problems, condylar resorption, and/or unfavorable genetic factors are a few. Because of its susceptibility to relapse [9], it is essential to evaluate more than the immediate posttreatment results when determining long-term stability [10–12].

Skeletal anchorage-supported systems [13] are used in more complex cases that otherwise would be impossible to treat unless they undergo orthognathic surgery [14, 15]. These temporary anchorage devices are used to facilitate tooth movements that were not predictably performed by traditional mechanotherapy, such as intrusion, distalization, and protraction of molars [16].

Some reviews [1, 13, 17] have been carried out to evaluate the effects of skeletal anchorage devices on molar intrusion for anterior open bite closure, but none of these reviews evaluated the medium and long-term stability of changes produced by these devices. Thus, the objective of this systematic review and meta-analysis is to assess the degree of stability of AOB treatment performed through the molar intrusion supported with skeletal anchorage at least 1 year after treatment completion.

## Material and methods

This systematic review was reported following PRISMA guidelines (www.prisma-statment.org) and registered on the National Institute of Health Research Database (www.crd.york.ac.uk/prospero, protocol: CRD 42016037513).

### Eligibility criteria

The following selection criteria were applied for the review:

1. Study design: randomized (RCT) or non-randomized clinical trials (non-RCT) including those studies without a control group evaluating only one type of intervention as case series or studies with before and after design.
2. Population: adolescent or adult patients with anterior open bite malocclusion undergoing treatment with permanent molar intrusion.
3. Intervention: patients that underwent orthodontic treatment for AOB correction by means of upper or lower molar intrusion supported by temporary skeletal anchorage were included.
4. Comparison: clinical studies comparing pre-treatment (T1), posttreatment (T2), and at least 1 year into retention (T3).
5. Outcome: the main outcome was to measure the stability of anterior open bite treatment after molar intrusion with skeletal anchorage assessed by cephalometric measurements. Angular and linear measures were used to evaluate the vertical changes of the mandible by the following: overbite and lower anterior facial height (LAFH). The stability of molar intrusion was assessed by the following: mandibular molar height (L6-MP) and the maxillary posterior dentoalveolar height (U6-PP). Secondary outcomes: maxillary incisor position (U1-PP) and mandibular incisor position (L1-MP).
6. Exclusion criteria: studies that include patients with craniofacial syndromes, abnormalities, cleft lip or palate, surgical treatment, patients in primary or mixed dentition, case reports, literature review, abstracts, discussions, and animal studies.

### Information sources

Several electronic databases (PubMed, Cochrane Library, ScienceDirect, Google Scholar, Scopus, Lilacs, OpenGrey, and Web of Science) were searched. A manual search was also performed over the references of the selected articles aiming to find articles that were not identified during the electronic database searches. In order to find unpublished or potentially relevant studies, experts in the area were contacted through e-mail. MetaRegister database of controlled clinical trials and Clinicaltrials.gov were also explored.

### Search strategy and study selection

No date or language restriction was imposed. Two reviewers (DSGE and PEOM) independently carried out the search until May 2020. Disagreements were resolved by discussion and consultation with the third and fourth authors (CFM and DN). The search strategy was performed using a combination of words (Additional file: 1) modifying the search terms and word combination as required in each database. The articles were imported into a reference manager program (EndNote X7.0.1, Thomson Reuters) in order to remove duplicate studies.

### Data items and collection

Two authors (DSGE and PEOM) independently extracted characteristics and outcomes from the included studies, summarizing the following items: type of study, study design, participants, measures investigated, treatment type, treatment time, and stability degree (Table 1).

**Table 1:** Characteristics of the studies included in the current systematic review

Article author/year	Participants		Methods	Treatment time/follow-up time	Rated outcomes	Treatment performed/study material	Results				Conclusion
	Sample source	Patients (n)/mean age (SD) in years									
							Statistical analysis	Mandibular rotation/Applied force	Effects on facial morphology	Side effects	
**Scheffler et al/2014** [18]	Private practice. Boone, NC	30/24.1 (± 10.7) years	Lateral cephalometric analysis	Intrusion period, 3.6–9.6 months Total treatment period, 6–33 months	Overbite, Go-Gn/Sn, LAFH, U6-pp, L6-GoGn. U1-PP. L1-MP	Pre and post treatment: miniplate, mini-implants and acrylic plate L-shaped miniplate in the upper-back region	Multivariate regression	Counterclockwise rotation: decrease of Go-Gn/Sn. After the 1st and 2nd year of treatment: there were changes/150 g per side	Decreased LAFH (1 mm) after 1 and 2 years of follow-up.	Lower molar extrusion, elongation of lower and upper incisors. Fifteen and 22% of patients: recurrence after 1 and 2 years of follow-up	Posterior and upper intrusion may be satisfactory in moderately severe cases, but relapse (0.5 to 1.5 mm) is likely to occur.
**Baek et al./2010** [15]	Department of Orthodontics, Dental Hospital of Yonsei, Korea	9/ 23.7 years	Lateral cephalometric analysis	Treatment period 28.8 months. Mini-implants 5.4 months. Retention 41 months	Overbite,SN-Go Me, AFH,U6-PP, L1-MP, U1-PP,	Exodontia of premolars. Molar intrusion: mini-implants + elastomeric chain + rigid transpalatal arch	Shapiro-Wilk;THE NEW; Pearson’s correlation	Counterclockwise rotation: anterior facial height decreased by 2.53 mm/NR	Decreased AFH (2.53 mm), stable profile after 1 year of treatment.	22.88% of the vertical distance between maxillary molars and palatal plane and 17% of overbite after 3 years of treatment.	If an appropriate retention method is applied during the 1st year of treatment, posttreatment stability is guaranteed.
**Sugawara et al./2002** [14]	Department of Orthodontics Tohoku University, Japan	9 / 21.1 years	Lateral cephalometric analysis. Panoramic RX. Dental analysis	14.9 months/12 months	Overbite, MP/FH, LAFH, U6-PP, L6-MP. L1-MP, U1-PP	L-shaped Miniplate (SAS) in the lower molars. Orthodontic micro screws + force application with elastic modules	Paired *t* test	Counterclockwise rotation: FH/MP decreased by 1.3°/FH/MP increased by 0.4° after 1 year of follow-up/NR	Decreased ALFH and lip opening. Anteroposterior mandibular relationship better. Stable profile after 1 year of follow-up	27.2% of relapse in the first molars and 30.3% in second molars	SAS is effective for the treatment of open bite. Overcorrection is required
**Deguchi et al./2011** [19]	Tohoku University, Japan	30/24.3 (± 5.9)	Lateral cephalometric analysis	Treatment time:3 years and 2 years of follow-up	Overbite, MP/SN, U6-PP, L6-MP, LAFH. L1-MP,U1-PP	Miniimplants: exo of anterior premolars and elastics, sharp curve arches or edgewise multiloop technique. Sectional steel bows of 0.016 × 0.022. Extraoral arch of high pull	Mann Whitney, Wilcoxon	Counterclockwise rotation: 3° of increase of mandibular plane angle and 3 mm of increase in the vertical length of N-Me./NR	Significant difference between T1 and T2 in the labial protrusion,decreased in facial convexitybut group IA had the greatest change.	22% of superior molar relapse and 13% overbite after 2 years in the intervention group (AI)	The group with mini-implants presented greater relapse
**Marzouk et al./2015** [20]	Department of Orthodontics, Alexandria University, Egypt	26/ 22.5 years	Lateral cephalometric analysis	Intrusion period, 5–10 (months) Treatment period, 24–28 (months)	Overbite, N-S-Gn, U6-pp, L6-MP,L1-MP, U1-PP	Miniplate and upper modified Hawley plate. Titanium miniplate fixed with three screws and double transpalatal bar	test; intraclass correlation coefficient, analysis of variance, Pearson’s correlation	Counterclockwise rotation: overbite correction / 450 g per side	N-Me decreased 3.64 mm after 4 years of treatment. Decreased facial convexity of 2.36° and stability after 4 years.	Relapse of 10.20% in the upper molars in the 1st year after treatment and 13.37% 4 years after treatment.	Molar intrusion with zygomatic miniplates appears to be stable 4 years after treatment.
**Lee et al/2008** [21]	Department of Orthodontics, Yonsei University, Korea	11/ 23.3 years	Lateral cephalometric analysis	Treatment period 5.4 (months) Average maintenance period 17.4 months	MeGo/SN, Overbite, AFH, U6-PP, L1-MP, U1-PP	Mini-implants + elastomeric chain, transpalatal bar for molar intrusion	Descriptive statistics, paired *t* test, and Pearson’s correlation	Counterclockwise rotation, anterior facial decrease. Increased facial height of 0.18° after 17.4 months/NR	AFH decrease, mov. of Pogônio 2.17 mm. AFH increase after 17, 4 months in contention period	10.36% relapse for molar intrusion. 18.10% for overbite after 17.4 months	Upper molar intrusion with mini screws in adults is efficient for open bite correction with good stability.

*NR* no reported

### Quality assessment in individual studies

The JBI (Joanna Briggs Institute critical assessment tools) for the case series [22] was used to assess the methodological quality of the full-text articles using standardized critical assessment instruments that were specific to the type of research design used in eligible studies. Through this process, it is possible to identify the sources of bias by using criteria that the reviewers qualified with answers such as follows: yes, no, uncertain, or not applicable. We calculated the prevalence of “yes” scores (number of “yes”/number of articles) for each individual evaluation question. Two researchers (DSGE and PEOM) independently analyzed each criterion of the JBI tool. A third researcher and fourth researchers (CFM and DN) were consulted in case of disagreements.

The GRADE [23] (Grading of Recommendations Assessment, Development and Evaluation—https://gradepro.org/) was used to assess the degree of certainty of evidence in the included studies in the quantitative synthesis and meta-analysis. Some criteria were assessed to classify the results, such as study design, number of included studies, consistency of the results that evaluated the clinical differences, directness, bias reported, heterogeneity, and precision in the analysis of wide confidence intervals around the summary estimate. These criteria allowed the categorization of each cephalometric measurement, ranging from very low to high, according to the scores.

### Summary measures

Statistical analysis was performed through a meta-analysis of selected articles in quantitative synthesis using the Cochrane: Review Manager Software, version 5.3 (https://www.cochrane.org/). In this analysis, the following cephalometric measurements were included overbite, U1-PP, L1-MP, LFAH, U6-PP, and L6-MP. *I*^2^ tests for homogeneity were undertaken to quantify the extent of heterogeneity before each meta-analysis. *I*^2^ values above 50% would imply moderate to high heterogeneity and might preclude meta-analysis. For the interpretation of results, standardized mean differences and standard errors, *p* value (*p* < 0.05) was used. The standardized mean differences with 95% confidence intervals for each trial were calculated and combined using a random effects model, which was considered more appropriate in view of variations in samples, the different TADs used, and the different retention protocols. The representation of these results was done through forest plot employing the Review Manager Software, version 5.3 (https://www.cochrane.org/).

### Additional analyses

No additional analyses were performed due to limited adequate data.

## Results

### Study selection

After completing the electronic searches (Additional file: 1), 818 articles were identified, 181 in PubMed, 30 in the Web of Science, 56 in Scopus, 391 in Google Scholar, 77 in Cochrane, 1 in Lilacs, 9 in Clinical Trials, 1 in OpenGrey, and 72 in Science Direct. All duplicates were removed, leaving 734 articles.

According to the proposed eligibility criteria and analyzing the titles and abstracts, only 26 articles were considered as full text in phase 2. From them, 13 were later removed for not assessing stability, two were removed for assessing stability only for 6 months, one was removed for not using skeletal anchorage during treatment, one for not presenting the initial cephalometric values, one for using the same sample from another study selected, one for not assessing AOB, and one for having a sample with only three patients (Table 2) leaving only six studies [14, 15, 18–21] for qualitative and 4 [14, 15, 20, 21] for quantitative synthesis and meta-analysis. None of the finally included studies was a RCT.

**Table 2:** Excluded articles according to the eligibility criteria

Article’s name	Reason of exclusion
1- Skeletal anchorage system for open-bite correction. Umemori, M. Sugawara, J. Mitani, H. Nagasaka, H. Kawamura, H. (1999)	Study excluded for not evaluating stability.
2- Nonextraction treatment of an open bite with microscrew implant anchorage. Park, Hyo-Sang Kwon, Oh-Won Sung, Jae-Hyun. (2006)	Study excluded for not evaluating stability.
3- Open bite correction by intrusion of posterior teeth with miniscrews Park, Young-Chel Lee, Han-Ah Choi, Nak-Chun Kim, Doo-Hyung. (2008)	Study excluded for not evaluating stability.
4- Skeletal Class lll severe openbite treatment using implant Anchorage. Sakai, Yuichi Kuroda, Shingo Murshid, Sakhr A. Takano-Yamamoto, Teruko (2008)	Study excluded for not evaluating stability.
5- Correction of skeletal open bite with implant anchored molar/bicuspid intrusion. Sherwood, Keith. (2007)	Study excluded for not evaluating stability.
6- Closing anterior open bites by intruding molars with titanium miniplate Anchorage Sherwood, Keith H. Burch, James G. Thompson, William J. (2002)	Study excluded for not evaluating stability.
7- Differential molar intrusion with skeletal anchorage in open-bite treatment Paik, Cheol-Ho McComb, Ryan Hong, Christine Hong (2016)	Study excluded for not evaluating stability.
8- Maxillary molar intrusion with zygomatic anchorage in open bite treatment: lateral and oblique cephalometric evaluation. de Oliveira, T. F. M., Nakao, C. Y., Gonçalves, J. R., & Santos-Pinto, A. (2015)	Study excluded for not evaluating stability.
9- Retratamento de mordida aberta esquelética com intrusão dos molares superiores com mini-implantes Farret, Marcel Marchiori Farret, Milton Meri Benitez. (2013)	Study excluded for not present the initial cephalometric values.
10- Posterior impaction with orthodontic miniscrews for openbite closure and improvement of facial profile Kravitz, N. D. Kusnoto, B. (2007)	Study excluded for not evaluating stability.
11- Molar intrusion in the management of anterior openbite and ‘high angle’ class II malocclusions. Cousley, R. R. (2014)	Study excluded for not evaluating stability.
12- Microscrew anchorage in skeletal anterior open-bite treatment. Xun, C. L., X Zeng, X Wang. (2007)	Study excluded for not evaluating stability.
13- Open-bite closure by intruding maxillary molars with skeletal anchorage. Seres, L. and A. Kocsis. (2008)	Study excluded for not evaluating stability.
14- A estabilidade do tratamento compensatório da mordida aberta anterior no paciente adulto. Valarelli, F. P., Lemos, A. R. B., Silva, C. C. D., Paccini, J. V. C., & Valarelli, D. P. (2013)	Study excluded for not evaluating skeletal anchorage
15- A 10-year follow-up case-report following surgical-correction of anterior open bite. Lew, K. K. K. and H. S. Loh. (1991)	Study excluded for be a case-report
16-Effectiveness and stability of anterior open bite correction using temporary skeletal anchorage: comparison to surgical outcomes. Thesis of the University of North Carolina at Chapel Hill. Hull, J. T. (2009)	Study excluded for evaluating a mean of 6 months of stability
17- Dentoskeletal changes following mini-implant molar intrusion in anterior open bite patients. Hart, T. R., Cousley, R. R., Fishman, L. S., & Tallents, R. H. (2015)	Study excluded for not evaluating stability.
18- Treatment of severe anterior open bite with skeletal anchorage in adults: comparison with orthognathic surgery outcomes. Shingo Kuroda, Yuichi Sakai, Nagato Tamamura, Toru Deguchi, Teruko Takano-Yamamoto. (2007)	Study excluded for evaluating only 6 months of stability
19- Lateral open bite: treatment and stability Marise de Castro Cabrera, Carlos Alberto Gregório Cabrera, Karina Maria Salvatore de Freitas, Guilherme Janson, Marcos Roberto de Freitas.(2010)	Study excluded for not evaluating anterior open bite
20- Long-term stability of soft tissue changes in anterior open bite adults treated with zygomatic miniplate-anchored maxillary posterior intrusion. Marzouk ES, Kassem HE. (2018)	Study excluded for using the same sample from evaluation of long-term stability of skeletal anterior open bite correction in adults treated with maxillary posterior segment intrusion using zygomatic miniplates. Marzouk ES, Kassem HE (2016)

**Fig. 1:**
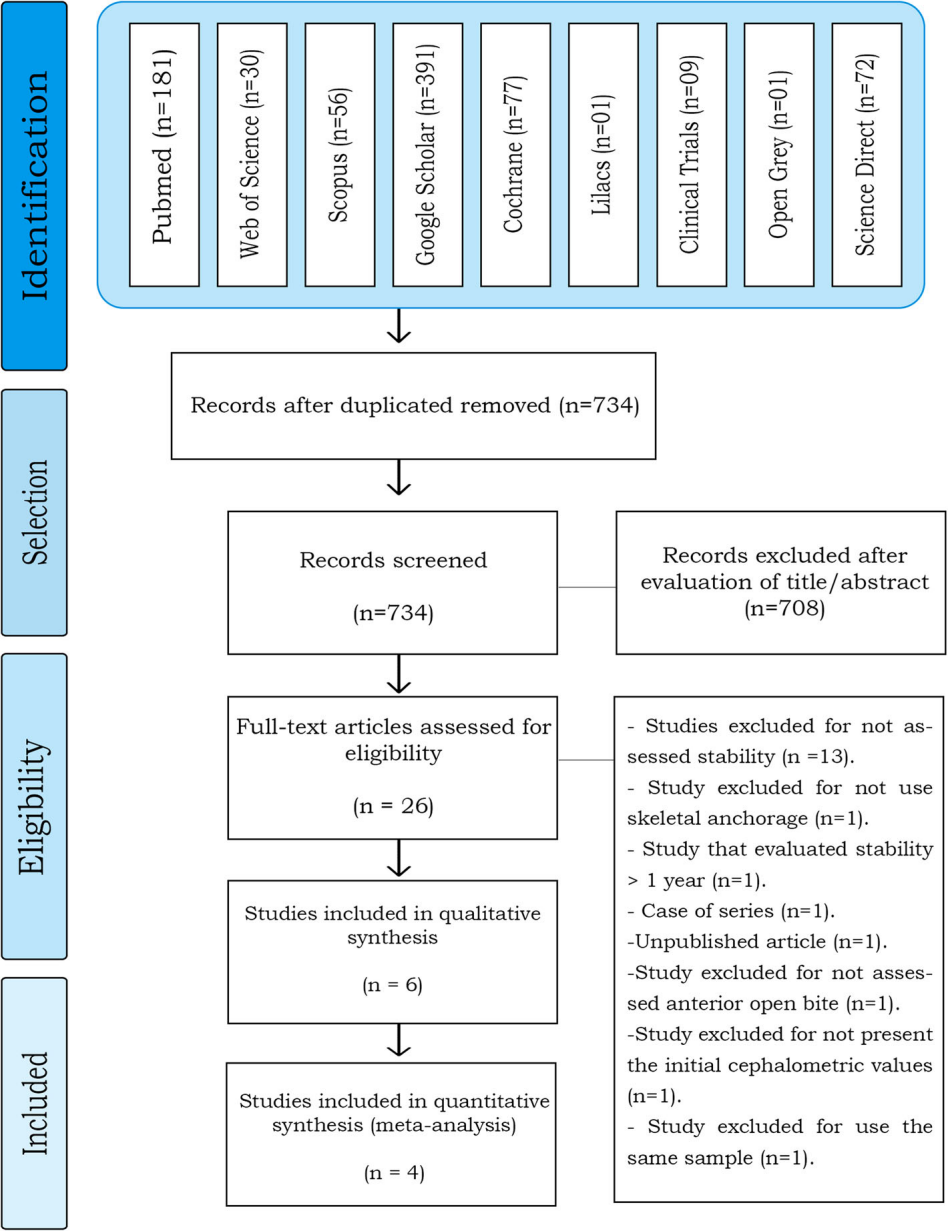
Flow diagram of literature search and selection criteria. Adapted for PRISMA

A flow diagram of the process of identification, inclusion, and exclusion of studies is presented in Fig.1.

### Study characteristics

A detailed description of the studies included in this systematic review is presented in Table 1. All articles selected reported the mean age of the patients, with a minimum of 21.1 years [14] and a maximum of 24.3 years [19].

Regarding the time of follow-up and sample characteristics, it was observed that two studies [14, 15] followed nine patients, but one of them [14] followed the patients for a period of 1 year—a sample of seven females and two males, while the other [15] followed patients for 3 years—a sample of eight females and 1 male. Another study [21] evaluated eleven patients for 17.4 months, not reporting their sex. Deguchi et al. [19] evaluated fifteen female patients for a period of 3 years. Two other studies [18, 20] presented a more robust sample. One [20] reported a sample of 24 patients followed for 4 years, while another [18] evaluated 30 patients, being this the only study that reported a dropout during the follow-up, ending with 25 patients after 2 years.

### Risk of bias within studies

The Joanna Briggs Institute critical appraisal tool for case series studies [22] was applied to the six non-randomized selected articles (Table 3), and was observed a prevalence of “Yes” scores for each individual appraisal question. Some confounding issues as limitations were identified in two articles [14, 18] for Q3 (question #3) and for three articles [15, 19, 20] for Q4 (question #4). All limitations of the included studies were listed in Table 3.

**Table 3:** The Joanna Briggs Institute critical appraisal checklist tool for case series studies applied to all of the included articles

JBI question	Sheffler et al. [18]	Baek et al. [15]	Sugawara et al. [14]	Deguchi et al. [19]	Marzouk et al. [20]	Lee et al. [21]
Q1. Were there clear criteria for inclusion in the case series?	U	Y	Y	Y	Y	Y
Q2. Was the condition measured in a standard, reliable way for all participants included in the case series?	Y	Y	Y	Y	Y	Y
Q3. Were valid methods used for identification of the condition for all participants included in the case series?	N	Y	N	Y	Y	Y
Q4. Did the case series have consecutive inclusion of participants?	Y	N	Y	N	N	Y
Q5. Did the case series have complete inclusion of participants?	Y	Y	Y	Y	Y	Y
Q6. Was there clear reporting of the demographics of the participants in the study?	Y	Y	Y	Y	Y	Y
Q7. Was there clear reporting of clinical information of the participants?	Y	Y	Y	Y	Y	Y
Q8. Were the outcomes or follow-up results of cases clearly reported?	U	Y	Y	Y	Y	Y
Q9. Was there clear reporting of the presenting site(s)/clinic(s) demographic information?	Y	Y	Y	Y	Y	Y
Q10. Was appropriate statistical analysis used?	Y	Y	Y	Y	Y	Y

*Y* yes, *N* no, *U* unclear

### Results of individual studies

To evaluate the stability of molar, overbite, mandibular rotation, and anterior facial height, the standardized mean and standard deviation of pre-treatment, posttreatment, and at least 1-year follow-up cephalometric values can be found in Table 4.

**Table 4:** Cephalometric measurements extracted from selected articles

Article	Outcome	Average pre-treatment	Mean after treatment	Difference of averages	Follow-up after 1 year	(%)	Follow-up ≥ 2 years	(%)	Follow-up ≥ 3 years	(%)
Scheffler et al./2014 [18]	Go-Gn/Sn Overbite LAFH U6-PP L6-Go-Gn L1-MP U1-PP	NR NR NR NR NR NR NR	NR NR NR NR NR NR NR	− 1.2 (1.0) 2.2 (1.6) − 1.6 (2.2) − 2.3 (1.4) 0.6 (1.6) NR 0.1 (3.8)	0.0 (0.9) − 0.3 (0.8) − 0.2 (1.4) 0.5 (1.1) − 0.6 (1.3) NR − 0.3 (1.0)	– – – – – – –	0.0 (0.8) − 0.4 (1.1) − 0.3 (1.4) 0.5 (1.2) − 0.3 (1.3) NR − 0.3 (1.2)	– – – – – – –	NR NR NR NR NR NR NR	– – – – – – –
Baek et al./2010 [15]	SN-Go Me Overbite AFH U6-PP L1-MP U1-PP	45.44 (4.11) − 3.91 (1.65) 133.95 (5.55) 26.88 (1.12) 43.58 (2.46) 31.50 (2.67)	43.41 (4.41) 1.65 (0.82) 131.41 (6.10) 24.50 (1.64) 45.17 (2.78) 32.56 (2.12)	− 2.03 (1.59) 5.56 (1.94) − 2.53 (1.90) − 2.39 (1.76) 1.59 (2.10) 1.05 (1.40)	43.68 (4.88) 0.66 (0.79) * 131.86 (5.54) 24.89 (1.69) * 44.90 (2.58) 32.49 (1.91)	(13%) (17%) (16%) (16%) (17%) (6%)	NR NR NR NR NR NR	– – – –	43.98 (4.76) 0.45 (1.09) * 132.32 (5.87) 24.94 (1.68) * 45.12 (2.57) 32.83 (2.15)	(28%) (21%) (35%) (18%) (3%) (26%)
Sugawara et al./2002 [14]	MP/FH Overbite LAFH L6-MP U6-PP L1-MP U1-PP	33.1 (2.1) − 2.8 (1.68) 76.1 (5.8) 35.7 (4.1) 34.0 (3.0) 44.5 (3.9) 29.8 (3.1)	31.7 (2.4) 2.1 (0.8) 74.6 (6.0) 33.9 (4.1) 25.0 (2.8) 45.8 (4.1) 30.9 (3.3)	− 1.3 (NR) 4.9 (NR) − 1.5 (NR) − 1.8 (NR) 1.0 (NR) 1.3 (NR) 1.1 (NR)	32.2 (3.0)* 1.2 (0.8)* 75.2 (5.8)* 34.2 (4.4)* 25.1 (2.5)* 45.3 (4.3) 30.7 (3.1)	(38%) (18%) (40%) (16%) (10%) (38%) (18%)	NR NR NR NR NR NR NR	– – – – – – –	NR NR NR NR NR NR NR	– – – – – – –
Deguchi et al./2011 [19]	Overbite MP/SN U6-PP L6-MP LAFH L1-MP U1-PP	− 4.4 (1.2) 45.8 (6.0) 26.9 (3.0) 36.0 (2.5) 74.7 (5.9) 46.3 (3.4) 32.4 (2.3)	1.8 (1.1) 42.2 (6.7) 24.6 (2.5) 35.2 (1.9) 72.2 (5.1) 46.6 (2.8) 33.4 (2.3)	6.2 (1.7) − 3.6 (2.1) − 2.3 (1.3) − 0.8 (1.3) − 2.6 (2.5) 0.3 (2.3) 1.0 (1.6)	NR NR NR NR NR NR NR	– – – – – – –	1.0 (0.9)* 43.8 (6.5)* 25.1 (2.8)* 37.0 (1.9)* 72.2 (5.1)* 46.3 (3.1) 33.4 (2.3)	(12%) (44%) (21%) (225%) (0%) (100%) (0%)	NR NR NR NR NR NR NR	– – – – – – –
Marzouk et al./2015 [20]	Overbite N-S-Gn U6-pp L6-MP L1-MP U1-PP	− 4.75 (2.27) 77.09 (3.01) 28.27 (2.55) 34.43 (1.27) 44.05 (2.79) NR	2.18 (0.48) 74.68 (2.67) 25.23 (2.14) 34.86 (1.35) 45.62 (2.82) NR	6.93 (1.99) − 2.23 (0.37) − 3.04 (0.79) 0.43 (0.53) 1.57 (0.07) NR	1.61 (0.42) 75.39 (2.83)* 25.54 (2.17)* 34.57 (1.13) 45.32 (2.80) NR	(8%) (31%) (10%) (67%) (19%) NR	NR NR NR NR NR NR	– – – – – –	1.41 (0.39) 75.69 (2.89) 25.64 (2.17) 34.29 (1.38) 45.56 (2.82) NR	(11%) (45%) (13%) (132%) (4%) NR
Lee et. al/2008 [21]	MeGo/SN Overbite AFH U6-PP L1-MP U1-PP	44.9 (1.7) − 3.7 (1.7) 133.4 (5.4) 26.7 (1.2) NR 31.30 (2.46)	42.9 (4.5) 1.7 (0.7) 130.8 (5.7) 24.5 (1.7) NR 32.30 (1.94)	3.9 (− 2.0) 5.4 (1.8) − 2.6 (1.9) − 2.2 (1.7) NR 1 (1.21)	43.0 (4.8)* 0.7 (0.7)* 131.1 (5.4)* 24.7 (1.6)* NR 32.25 (1.88)	(2.5%) (18%) (11%) (9%) NR (5%)	NR NR NR NR NR NR	– – – – – –	NR NR NR NR NR NR	– – – – – –

*NR* not reported

*p < 0.05

Three of the six included articles [14, 19, 21] used mini-implants exclusively as a skeletal anchorage, while two [14, 20] used miniplates only and another [18] applied both methods simultaneously.

Variability was observed in relation to the localization of the anchorage devices. One of the studies [18] installed the anchorage units bilaterally at the base of the zygomatic arch. Baek et al. [15] divided into two groups, one group only on the buccal side and another group on the buccal and palatine sites. In others two papers [19, 21], the devices had been installed between the second premolar and first molar or between first and second molars in the maxillary buccal region. Marzouk et al. [20] inserted the miniplates to the contour of the mandibular surface of each zygomatic buttress, while Sugawara et al. [14] were the only ones who used the anchoring devices in the mandibular region, being anchored between the first and second molars.

Another difference was observed when comparing the devices used during the retention phase. Sugawara et al. [14] reported using the skeletal anchorage device itself as anchorage, whereas Deguchi et al. [19] used occlusal stops in the mandibular molars or mini-mandibular implants, Scheffler et al. [18] reported the use of an occlusal cover, Baek et al. [15] used an active retainer created with buccal buttons, elastomeric alloys, and the mini-implant itself, and Marzouk et al*.* [20] adopted the use of maxillary and mandibular Hawley plates at day and a maxillary Hawley retainer with a posterior bite plane was to be worn during the night throughout the first year. In the second year posttreatment, the latter group used the maxillary Hawley plate with a posterior bite plane and the conventional mandibular Hawley plate only at night and from the third year, only one night per week, while Lee et al. [21] did not report the mechanism employed during the retention phase.

After a year of follow-up, five articles found a maximum relapse of 0.6 mm molar intrusion [14, 15, 18, 20, 21]. Deguchi et al. [19] found a relapse of 1.7 mm (21%), and Scheffler et al. [20] reported 0.5 mm between 1 and 2 years, while Baek et al*.* [15] and Marzouk et al*.* [20] reported non-significant changes after 3 and 4 years of follow-up, respectively. None of the papers showed a statistically significant difference when evaluating the stability of mandibular autorotation. Regarding the relapse of overbite, it ranged from 0.6 (17%) to 1.61 mm (8%) after 1 year of follow-up [14, 15, 20, 21], 0.1 mm (16%) after 2 years [20], 0.21 (21%) after 3 years [15], and 0.20 (11%) after 4 years [20], when compared with the changes that occurred in the previous year.

### Meta-analysis results

**Fig. 2:**
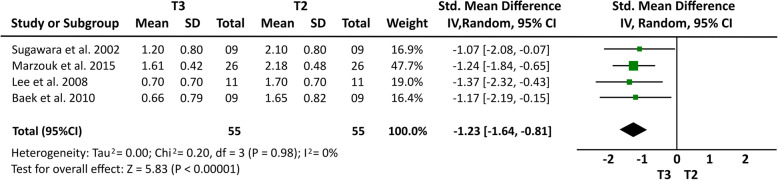
Forest plot of overbite difference between T2 and T3 in patients who used TADs for molar intrusion after 1-year follow-up. 95% confidence interval and 95% prediction interval

**Fig. 3:**
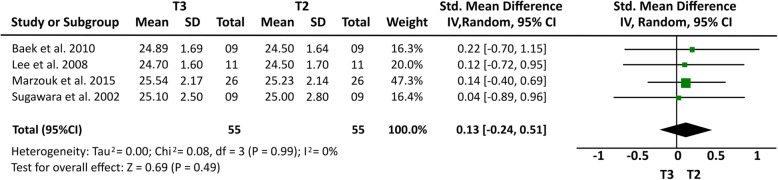
Forest plot U6-PP difference between T2 and T3 in patients who used TADs for molar intrusion after 1-year follow-up. 95% confidence interval and 95% prediction interval

**Fig. 4:**
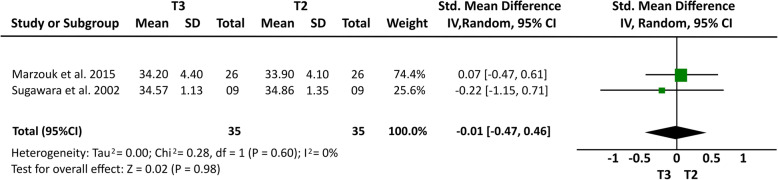
Forest plot of L6-MP difference between T2 and T3 in patients who used TADs for molar intrusion after 1-year follow-up. 95% confidence interval and 95% prediction interval

**Fig. 5:**
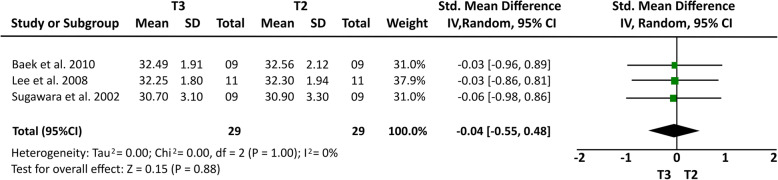
Forest plot of LAFH difference between T2 and T3 in patients who used TAD’s for molar intrusion after 1-year follow-up. 95% confidence interval and 95% prediction interval

**Fig. 6:**
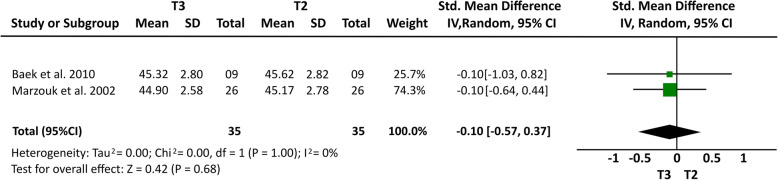
Forest plot U1-PP difference between T2 and T3 in patients who used TADs for molar intrusion after 1-year follow-up. 95% confidence interval and 95% prediction interval

**Fig. 7:**
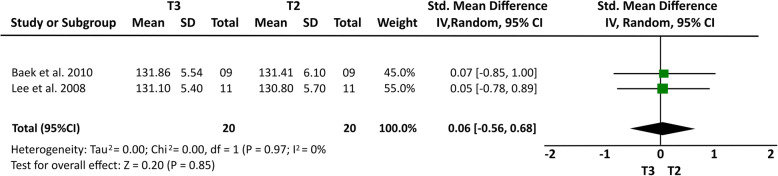
Forest plot of L1-MP difference between T2 and T3 in patients who used TADs for molar intrusion after 1-year follow-up. 95% confidence interval and 95% prediction interval

Some cephalometric values were analyzed through a meta-analysis of 4 articles [14, 15, 20, 21] that showed a greater homogeneity in relation to the measures studied and presented similar cephalometric values after 1-year follow-up. All four articles [14, 15, 20, 21] analyzed overbite and U6-PP. In a total sample of 55 patients, it was observed that overbite presented the higher relapse rate of − 1.23 mm (19% (95% CI − 1.64, − 0.81) Fig. 2) and U6-PP showed that the maxillary molar had a non-significant relapse, with a standardized mean difference of 0.13 mm (10% (95% CI − 0.24, 0.51) Fig. 3). When comparing these results with the L6-MP values obtained through two articles [14, 20] that analyzed 35 patients, we observed that the lower molars presented a non-significant lower mean relapse of − 0.01 (16% (95% CI − 0.47, 0.46) Fig. 4). It is necessary to consider the difference of sample sizes used for both measures. When analyzing incisor changes, through U1-PP among three articles [14, 15, 21], it was observed that the maxillary incisors presented a non-significant mean relapse of − 0.04 (18% (95% CI − 0.55, 0.48) Fig. 5). The results in mandibular incisors were similar when analyzing trough the L1-MP measure in a population of 35 patients obtained from two articles [15, 20], showing a non-significant relapse mean of − 0.10 (10% (95% CI − 0.57, 0.37) Fig. 6). The anterior facial height was analyzed in a sample of 20 patients obtained from 2 articles [15, 21], with a non-significant mean relapse of 0.06 mm, (11% (95% CI − 0.56, 0.68) Fig. 7). Statistical heterogeneity was observed in all performed meta-analyses (Figs. 2, 3, 4, 5, 6, and 7).

### Certainty level

The results obtained through the GRADE assessment [23] in relation to the meta-analysis results varied between low and very low certainty, due to “serious” limitations in imprecision since the significant heterogeneity in relation to the general result in these observational studies. These results also decreased especially by the inconsistency of the retention methods used by the four assessed articles. Heterogeneity was the main factor responsible for the limited quality of the evidence. The quality of clinical recommendations was upgraded due to the magnitude of relapse of the Overbite. All judgements made for the GRADE analysis of each outcome are presented in detail in Table 5.

**Table 5:** Cephalometric measurements extracted from selected articles

Article	Outcome	Average pre-treatment	Mean after treatment	Difference of averages	Follow-up after 1 year	(%)	Follow-up ≥ 2 years	(%)	Follow-up ≥ 3 years	(%)
Scheffler et al./2014 [18]	Go-Gn/Sn Overbite LAFH U6-PP L6-Go-Gn L1-MP U1-PP	NR NR NR NR NR NR NR	NR NR NR NR NR NR NR	− 1.2 (1.0) 2.2 (1.6) − 1.6 (2.2) − 2.3 (1.4) 0.6 (1.6) NR 0.1 (3.8)	0.0 (0.9) − 0.3 (0.8) − 0.2 (1.4) 0.5 (1.1) − 0.6 (1.3) NR − 0.3 (1.0)	– – – – – – –	0.0 (0.8) − 0.4 (1.1) − 0.3 (1.4) 0.5 (1.2) − 0.3 (1.3) NR − 0.3 (1.2)	– – – – – – –	NR NR NR NR NR NR NR	– – – – – – –
Baek et al./2010 [15]	SN-Go Me Overbite AFH U6-PP L1-MP U1-PP	45.44 (4.11) − 3.91 (1.65) 133.95 (5.55) 26.88 (1.12) 43.58 (2.46) 31.50 (2.67)	43.41 (4.41) 1.65 (0.82) 131.41 (6.10) 24.50 (1.64) 45.17 (2.78) 32.56 (2.12)	− 2.03 (1.59) 5.56 (1.94) − 2.53 (1.90) − 2.39 (1.76) 1.59 (2.10) 1.05 (1.40)	43.68 (4.88) 0.66 (0.79) * 131.86 (5.54) 24.89 (1.69) * 44.90 (2.58) 32.49 (1.91)	(13%) (17%) (16%) (16%) (17%) (6%)	NR NR NR NR NR NR	– – – –	43.98 (4.76) 0.45 (1.09) * 132.32 (5.87) 24.94 (1.68) * 45.12 (2.57) 32.83 (2.15)	(28%) (21%) (35%) (18%) (3%) (26%)
Sugawara et al./2002 [14]	MP/FH Overbite LAFH L6-MP U6-PP L1-MP U1-PP	33.1 (2.1) − 2.8 (1.68) 76.1 (5.8) 35.7 (4.1) 34.0 (3.0) 44.5 (3.9) 29.8 (3.1)	31.7 (2.4) 2.1 (0.8) 74.6 (6.0) 33.9 (4.1) 25.0 (2.8) 45.8 (4.1) 30.9 (3.3)	− 1.3 (NR) 4.9 (NR) − 1.5 (NR) − 1.8 (NR) 1.0 (NR) 1.3 (NR) 1.1 (NR)	32.2 (3.0)* 1.2 (0.8)* 75.2 (5.8)* 34.2 (4.4)* 25.1 (2.5)* 45.3 (4.3) 30.7 (3.1)	(38%) (18%) (40%) (16%) (10%) (38%) (18%)	NR NR NR NR NR NR NR	– – – – – – –	NR NR NR NR NR NR NR	– – – – – – –
Deguchi et al./2011 [19]	Overbite MP/SN U6-PP L6-MP LAFH L1-MP U1-PP	− 4.4 (1.2) 45.8 (6.0) 26.9 (3.0) 36.0 (2.5) 74.7 (5.9) 46.3 (3.4) 32.4 (2.3)	1.8 (1.1) 42.2 (6.7) 24.6 (2.5) 35.2 (1.9) 72.2 (5.1) 46.6 (2.8) 33.4 (2.3)	6.2 (1.7) − 3.6 (2.1) − 2.3 (1.3) − 0.8 (1.3) − 2.6 (2.5) 0.3 (2.3) 1.0 (1.6)	NR NR NR NR NR NR NR	– – – – – – –	1.0 (0.9)* 43.8 (6.5)* 25.1 (2.8)* 37.0 (1.9)* 72.2 (5.1)* 46.3 (3.1) 33.4 (2.3)	(12%) (44%) (21%) (225%) (0%) (100%) (0%)	NR NR NR NR NR NR NR	– – – – – – –
Marzouk et al./2015 [20]	Overbite N-S-Gn U6-pp L6-MP L1-MP U1-PP	− 4.75 (2.27) 77.09 (3.01) 28.27 (2.55) 34.43 (1.27) 44.05 (2.79) NR	2.18 (0.48) 74.68 (2.67) 25.23 (2.14) 34.86 (1.35) 45.62 (2.82) NR	6.93 (1.99) − 2.23 (0.37) − 3.04 (0.79) 0.43 (0.53) 1.57 (0.07) NR	1.61 (0.42) 75.39 (2.83)* 25.54 (2.17)* 34.57 (1.13) 45.32 (2.80) NR	(8%) (31%) (10%) (67%) (19%) NR	NR NR NR NR NR NR	– – – – – –	1.41 (0.39) 75.69 (2.89) 25.64 (2.17) 34.29 (1.38) 45.56 (2.82) NR	(11%) (45%) (13%) (132%) (4%) NR
Lee et. al/2008 [21]	MeGo/SN Overbite AFH U6-PP L1-MP U1-PP	44.9 (1.7) − 3.7 (1.7) 133.4 (5.4) 26.7 (1.2) NR 31.30 (2.46)	42.9 (4.5) 1.7 (0.7) 130.8 (5.7) 24.5 (1.7) NR 32.30 (1.94)	3.9 (− 2.0) 5.4 (1.8) − 2.6 (1.9) − 2.2 (1.7) NR 1 (1.21)	43.0 (4.8)* 0.7 (0.7)* 131.1 (5.4)* 24.7 (1.6)* NR 32.25 (1.88)	(2.5%) (18%) (11%) (9%) NR (5%)	NR NR NR NR NR NR	– – – – – –	NR NR NR NR NR NR	– – – – – –

*NR* not reported

*p < 0.05

## Discussion

Conventional molar intrusion can be performed with greater efficiency using skeletal anchorage devices, which require minimal or no patient collaboration [24]. Such approach provides an alternative to orthognathic surgery [14], which, in turn, can be associated with greater postoperative discomfort, has a higher financial cost, and could result in hospitalization and longer postsurgical rehabilitation times [8]. Furthermore, the risk of relapse after orthognathic surgery is relevant [22]. Although there are other orthodontic therapies, such as orthodontic treatments with and without extractions, previous studies report a relapse of 25.8% for open bite treatment with extractions [25] compared to 38.1% for cases without extraction. Besides relapse changes, these procedures require a longer treatment time [9, 26] compared to those supported by skeletal anchorage treatment.

The literature reports that the use of skeletal anchorage devices to perform this type of intrusion can cause a counterclockwise rotation of the mandible, improving facial esthetics [14, 15, 18–21]. There are sufficient articles that assess the effects of treatment using skeletal anchoring devices, including a systematic review [17]; however, few studies have evaluated the medium- and long-term stability of open bite treatment through molar intrusion using skeletal anchorage. No paper has examined this issue in a randomized design. Prospective studies are important to properly evaluate dropouts. Since only retrospective studies were included in this systematic review, cases with relapse may have been retreated and, therefore, not included in the analysis of primary studies. Thus, the findings of these can studies can underestimate the relapse amount.

Although all of the articles selected in this study have used skeletal anchorage to perform molar intrusion, there were differences in the positioning of these devices. Some inserted the mini-implants into the buccal and palatal region [15, 19, 21], while others only in the buccal region [14, 15, 18, 20]. Different auxiliary treatments were used combined with the skeletal anchorage devices, such as an association of mini-implants with elastomeric chains and transpalatal bars [21], mini-implants with extractions of premolars and extraoral appliances with high pull by using a power chain or ligature wires from the mini-implant to the sectional archwire [19], mini-implants with extractions and rigid transpalatal arch [15], miniplates with elastic modules [14], miniplates with Hawley, modified Hawley plates and transpalatal bars [20], and mini-implants and miniplates combined with acrylic plates [18].

In all included studies, open bite correction occurred due to maxillary incisor extrusion and molar intrusion, associated with a counterclockwise rotation of the mandible. When the treatment had been completed, different significant side effects were observed after 1 year of follow-up, such as first and second molar extrusion [20, 23] and increased overbite [14, 15, 18, 21]. However, in addition to the previously reported effects, it has been observed that there is a smallest vertical relapse of the maxillary (18%) [14, 15, 21] and mandibular incisors [15, 20], after 1 year of follow-up.

The meta-analysis showed a minimum relapse of maxillary and mandibular molar intrusion, mainly after the first year of follow-up. These values tend to increase over the years; hence, more effective methods of retention need to be applied in the first year posttreatment and after that. This analysis additionally suggested the existence of a small mean difference in the cephalometric measures that evaluating the molars’ relapse, but we have to considerer a difference in the sample size included in both meta-analyses, also the use of different skeletal anchorage devices and retention protocols. In addition, it is necessary to observe other factors that influence the period of retention aiming to decrease the relapse level reported in the literature [15]. The meta-analysis provided an idea of changes during a short-term follow-up period. However, the stated methodological heterogeneity among the included studies could result in criticism of applying a meta-analysis. Before and after studies [27] (case series) have significant risk of bias. Hence, the summaries should be considered with caution. Furthermore, no RCT was identified.

When analyzing the stability of molar intrusion, a greater relapse of 27.2% was observed for first mandibular molars and 30.3% at the second lower molars after 1 year of follow-up [14]; however, a greater stability was observed for the maxillary molars after 1 year of follow-up, showing a rate of relapse around 12% [18, 20, 21], which showed a tendency to increase in the second year posttreatment, with values ranging between 13 [20] and 21 [19]. After 3 years, posttreatment values were 18% [14] for relapse, with 80% of these changes occurring during the first year posttreatment. Overall, these relapse values give a success rate of 77% after 3 years of follow-up, which is similar to that observed after orthognathic surgery, which ranged from 79% after 3 years [28] to 85% after 5 years of follow-up [29].

Lee et al. [21] showed an overbite relapse of 18% in patients after 1 year posttreatment; while Deguchi et al. [19] and Scheffler et al. [18] reported a relapse of 16 to 12%, respectively. Marzouk et al. [20] reported a relapse rate of 11% after 4 years of treatment. Regarding conventional orthodontic treatment for anterior open bite, the literature reports that there is a 30% relapse after 10 years of follow-up [9], corroborating the results found by Deguchi et al. [19] who reported the same percent of instability after 2 years of follow-up.

Concerning to the skeletal anchorage devices, the lowest rate of overbite relapse after 1 year of follow-up was observed in patients who received treatment with upper miniplates combined with acrylic plates or transpalatal bars [18, 20], while the highest amount of changes was found in patients who received treatment with L-shaped miniplates in the mandibular cortical bone [18]. The latter findings can be explained due to the different bone densities between maxilla and mandible [30].

After the first year of follow-up, the values obtained in the mandibular rotation counterclockwise tend to decrease [14, 18], suggesting that there is a clockwise rotation of the mandible in the long term in these samples. Regarding the morphological effects obtained, there was a decrease in anterior facial height and facial convexity in all papers evaluated [14, 15, 18, 20, 21], except for Deguchi et al. [19] who did not cite this information. Among these effects, a decrease was reported in the labial opening along with an improvement of the anteroposterior mandibular relation [14, 19].

### Limitations of the available evidence

This systematic review and meta-analyses presented some limitations, including the presence of few primary articles that followed the stability of molar intrusion in long term, after three or more years. Furthermore, none of the included papers have examined a control group, even with a different treatment approach for comparison. Although the techniques employed in the selected studies used different auxiliary devices, it should be kept in mind that many therapies might be combined in orthodontics to arrive to the same result. Furthermore, no randomized clinical trial was found.

Due to the limited evidence of the articles selected in this systematic review and meta-analyses, it is suggested that controlled clinical randomized studies with larger samples and long-term follow-up are necessary for a more reliable estimation of the stability of anterior open bite when treated by intrusion of the posterior teeth by means of skeletal anchorage compared to other treatment modalities. Prospective studies are important to properly evaluate dropouts. When only retrospective studies are included, cases with relapse may have been retreated and, therefore, excluded from the posttreatment evaluation.

As mentioned before, the justification for the meta-analysis is questionable due to the significant methodological heterogeneity in the skeletal anchorage approaches and retention protocols. Here, this approach is used to calculate a gross estimation of effect size. This estimation is associated with a large uncertainty level.

## Conclusion

The stability of open bite treatment through molar intrusion using skeletal anchorage in adult patients can be considered relatively unstable since 10 to 30% of relapse occurs in both molars. These relapse levels are relatively similar even when differences in skeletal anchorage approaches and retention protocols are considered. The level of certainty off the meta-analysis results ranged from very low to low.

This review also showed a progressive relapse after the first year post-treatment; therefore, more effective methods of retention should be maintained in the long-term follow-up.

## Supplementary information

**Additional file 1. **Search strategy

## Data Availability

The datasets used and/or analyzed during the current study are available from the corresponding author on reasonable request.

## Abbreviations

AOB: Anterior open bite
T1: Pre-treatment
T2: Posttreatment
T3: At least 1 year into retention
LAFH: Lower anterior facial height
L6-MP: Mandibular molar height
U6-PP: Maxillary posterior dentoalveolar height
U1-PP: Maxillary incisor position
L1-MP: Mandibular incisor position
ROBINS-I tool: Risk of Bias in Non-randomized Studies of Interventions
ROB: Risk of bias
GRADE: Grading of Recommendations Assessment, Development and Evaluation
TAD: Temporary anchorage device
RCT: Randomized controlled trial
