# Supplementary material for: Stability of anterior open bite treatment with molar intrusion using skeletal anchorage: a systematic review and meta-analysis
Source: Prog Orthod. 2020 Sep 5;21:35. doi: 10.1186/s40510-020-00328-2 (PMC7474024; doi:10.1186/s40510-020-00328-2)
Supplement: Supplementary file 1 — Additional file 1. Search strategy [file 40510_2020_328_MOESM1_ESM.docx]

Table 1: Search strategy

| **DATABASE** | **SEARCH STRATEGY** |
| --- | --- |
| **PUBMED** | P= Search (((((adult[MeSH Terms]) OR adult[Title/Abstract]) OR adults[Title/Abstract])) OR ((Adolescent[MeSH Terms]) OR Adolescent[Title/Abstract])) AND ((((((Open bite[MeSH Terms]) OR Open bite[Title/Abstract]) OR Bite, Open[Title/Abstract]) OR Nonocclusion[Title/Abstract]) OR Openbite[Title/Abstract]) OR Apertognathia[Title/Abstract]) |
|  | I= Search (((((((((((((((Orthodontic anchorage procedure[MeSH Terms]) OR Orthodontic anchorage procedure[Title/Abstract]) OR Anchorage Procedure, Orthodontic[Title/Abstract]) OR Anchorage Procedures, Orthodontic[Title/Abstract]) OR Orthodontic Anchorage Procedure[Title/Abstract]) OR Procedure, Orthodontic Anchorage[Title/Abstract]) OR Procedures, Orthodontic Anchorage[Title/Abstract]) OR Orthodontic Anchorage Techniques[Title/Abstract]) OR Anchorage Technique, Orthodontic[Title/Abstract]) OR Anchorage Techniques, Orthodontic[Title/Abstract]) OR Orthodontic Anchorage Technique[Title/Abstract]) OR Technique, Orthodontic Anchorage[Title/Abstract]) OR Techniques, Orthodontic Anchorage[Title/Abstract])) OR (((((Mini implant[Title/Abstract]) OR Mini plates[Title/Abstract]) OR Mini screw[Title/Abstract]) OR TAD´s[Title/Abstract]) OR Temporary anchorage devices[Title/Abstract])) OR Molar intrusion[Title/Abstract] |
|  | P+I |
| **WEB OF SCIENCE** | TÓPICO: (adult*) OR TÓPICO: ("adults") OR TÓPICO: (adolescent*) OR TÓPICO: ("adolescents") AND TÓPICO: (Open bite*) OR TÓPICO: ("Bite, Open") OR TÓPICO: ("Nonocclusion") OR TÓPICO: ("Openbite") OR TÓPICO: ("Apertognathia") AND TÓPICO: ("Mini implant") OR TÓPICO: ("Mini plates") OR TÓPICO: ("Mini screw") OR TÓPICO: ("TADs") OR TÓPICO: ("Temporary anchorage devices") |
| **SCOPUS** | P= ( ( ( TITLE-ABS-KEY ( adult* )  OR  TITLE-ABS-KEY ( "adults" ) ) )  OR  ( ( TITLE-ABS-KEY ( adolescent* )  OR  TITLE-ABS-KEY ( "adolescents" ) ) ) )  AND  ( ( TITLE-ABS-KEY ( open  AND bite* )  OR  TITLE-ABS-KEY ( "Bite, Open" )  OR  TITLE-ABS-KEY ( "Nonocclusion" )  OR  TITLE-ABS-KEY ( "Openbite" )  OR  TITLE-ABS-KEY ( "Apertognathia" ) ) ) |
|  | I= ( ( TITLE-ABS-KEY ( orthodontic  AND anchorage  AND procedure* )  OR  TITLE-ABS-KEY ( "Orthodontic anchorage procedure" )  OR  TITLE-ABS-KEY ( "Anchorage Procedure, Orthodontic" )  OR  TITLE-ABS-KEY ( "Anchorage Procedures, Orthodontic" )  OR  TITLE-ABS-KEY ( "Orthodontic Anchorage Procedure" )  OR  TITLE-ABS-KEY ( "Procedure, Orthodontic Anchorage" )  OR  TITLE-ABS-KEY ( "Procedures, Orthodontic Anchorage" )  OR  TITLE-ABS-KEY ( orthodontic  AND anchorage  AND techniques )  OR  TITLE-ABS-KEY ( "Anchorage Technique, Orthodontic" )  OR  TITLE-ABS-KEY ( "Anchorage Techniques, Orthodontic" )  OR  TITLE-ABS-KEY ( "Orthodontic Anchorage Technique" )  OR  TITLE-ABS-KEY ( "Technique, Orthodontic Anchorage" )  OR  TITLE-ABS-KEY ( "Techniques, Orthodontic Anchorage" ) ) )  OR  ( ( TITLE-ABS-KEY ( "Mini implant" )  OR  TITLE-ABS-KEY ( "Mini plates" )  OR  TITLE-ABS-KEY ( "Mini screw" )  OR  TITLE-ABS-KEY ( "TAD´s" )  OR  TITLE-ABS-KEY ( "Temporary anchorage devices" ) ) )  OR  ( TITLE-ABS-KEY ( "Molar intrusion" ) ) View Less |
|  | P+I |
| **Google Scholar** | Adult OR Adolescents+"open bite"+"molar intrusion"+"orthodontic anchorage procedures" OR "TAD's" OR "temporary anchorage devices"+stability |
| **Cochrane** | P= (Adults$):ti,ab,kw OR (Adult):ti,ab,kw OR (Adolescent$):ti,ab,kw OR (Adolescents):ti,ab,kw AND (Open bite$):ti,ab,kw OR ("Bite, Open"):ti,ab,kw OR (Nonocclusion):ti,ab,kw OR (Openbite):ti,ab,kw OR (Apertognathia):ti,ab,kw |
|  | I= (Orthodontic anchorage procedure$):ti,ab,kw OR ("Anchorage Procedure, Orthodontic"):ti,ab,kw OR ("Anchorage Procedures, Orthodontic"):ti,ab,kw OR ("Orthodontic Anchorage Procedure"):ti,ab,kw OR ("Procedure, Orthodontic Anchorage" OR "Orthodontic Anchorage Techniques" OR "Anchorage Technique, Orthodontic" OR "Anchorage Techniques, Orthodontic" OR "Orthodontic Anchorage Technique" OR "Technique, Orthodontic Anchorage" OR "Techniques, Orthodontic Anchorage"):ti,ab,kw OR ("molar intrusion"):ti,ab,kw OR ("Mini implant"):ti,ab,kw OR ("Mini plates"):ti,ab,kw OR ("Mini screw"):ti,ab,kw OR ("TAD´s"):ti,ab,kw OR ("Temporary anchorage devices"):ti,ab,kw |
|  | C= (Denture stability$):ti,ab,kw OR ("Retention, Denture"):ti,ab,kw OR ("Denture Stability"):ti,ab,kw OR ("Stability, Denture"):ti,ab,kw OR (Relapse):ti,ab,kw OR ("Post retention"):ti,ab,kw OR (Stability):ti,ab,kw OR ("Follow up"):ti,ab,kw |
|  | P+I+C |
| **Lilacs** | Adults and Open Bite and Molar Intrusion |
| **Clinical Trials** | Adults and Anterior open bite |
| **Science Direct** | Keywords: ("adults" OR "adolescents") AND “Open Bite” AND “Molar Intrusion” AND (“Orthodontic anchorage devides” OR “temporary anchorage devices” OR TAD’s OR "Miniscrew") AND ("Stability" OR "Relapse" OR "post retention")) |
| **Open Grey** | Adults and Open bite |
